# Development and validation of competitive risk model for older women with metaplastic breast cancer

**DOI:** 10.1186/s12905-023-02513-x

**Published:** 2023-07-14

**Authors:** Jie Tang, Dianlong Zhang, Xiudan Pan

**Affiliations:** 1 Department of Biostatistics and Epidemiology, Public Health School, Shenyang Medical College, Huanghe North Street 146, Shenyang, 110034 China; 2 Women and Children’s Hospital, Qingdao University, 6 Tongfu Road, Shibei District, Qingdao, 266000 China

**Keywords:** Competitive risk model, Older women, Metaplastic breast cancer, SEER

## Abstract

**Background:**

Metaplastic breast cancer (MpBC) is a rare histological subtype of breast cancer. This study aims to establish a competitive risk model for older women with MpBC to predict patients’ survival accurately.

**Methods:**

Data on patients diagnosed with MpBC from 2010 to 2019 are from the Surveillance, Epidemiology and End Results (SEER) program in the United States. All patients were randomly assigned to the training set and validation set. The proportional sub-distribution risk model was used in the training set to analyze the risk factors affecting patient death. Based on the risk factors for cancer-specific mortality (CSM) in patients, we constructed a competitive risk model to predict patients’ 1-, 3-, and 5-year cancer-specific survival. Then we used the concordance index (C-index), the calibration curve and the area under the receiver operating characteristic curve (AUC) to validate the discrimination and accuracy of the model.

**Results:**

One thousand, four hundred twelve older women with MpBC were included in this study. Age, T stage, N stage, M stage, tumor size, surgery and radiotherapy were risk factors for CSM. We established a competitive risk model to predict 1-, 3-, and 5-year cancer-specific survival in older women with MpBC. The C-index of the model was 0.792 in the training set and 0.744 in the validation set. The calibration curves in the training and validation sets showed that the model’s predicted values were almost consistent with the actual observed values. The AUC results show that the prediction model has good accuracy.

**Conclusion:**

We developed a competitive risk model based on these risk factors to predict cancer-specific survival in older women with MpBC. The validation results of the model show that it is a very effective and reliable prediction tool. This predictive tool allows doctors and patients to make individualized clinical decisions.

**Supplementary Information:**

The online version contains supplementary material available at 10.1186/s12905-023-02513-x.

## Introduction

Breast cancer is the most common cancer in women. Metaplastic breast cancer (MpBC) is a rare histological subtype, accounting for 0.25–2% of breast cancers [1]. MpBC has a poor clinical outcome and is more aggressive than invasive ductal carcinoma [2]. MpBC has been considered a unique pathological diagnosis since 2000, and its incidence has increased [3]. MpBC shows rapid tumour growth at diagnosis, with a very high risk of progression and recurrence [4]. Due to its rarity, the pathogenesis, treatment, and clinical outcome of MpBC remain unclear. In addition, MpBC has typical chemotherapy drug resistance, and chemotherapy plays a limited role in treating MpBC and preventing disease progression [5]. These factors lead to poor prognosis and low survival rate of MpBC. Therefore, accurate prediction of the prognosis of MpBC patients can help doctors and patients to provide helpful information and individualized treatment.

Breast cancer is widespread in older women. However, because of the lack of standard treatment for older breast cancer patients, undertreatment leads to higher rates of breast cancer recurrence and mortality [6, 7]. Older women and their families face multiple barriers when choosing treatment, including prejudices such as the inability to tolerate treatment, failure to benefit from treatment, and unworthy treatment. In addition, older breast cancer patients are often excluded from clinical trials, or only a few healthy older women receive clinical trials [8]. Therefore, the clinical data of older women with breast cancer are often lacking, so sufficient data cannot be obtained to guide treatment. Although breast cancer in older women appears to be a biologically favourable tumor (hormone receptor-positive), it tends to be larger, and lymph node-positive [9], and hormone receptors are primarily negative in older women with MpBC. Older women are also accompanied by various comorbidities and cognitive decline [10, 11]. Therefore, older women with breast cancer need more individualized treatment.

The nomogram is a simple and reliable predictive tool that can predict patient survival based on risk factors affecting patient survival [12]. Various predictive tools have been developed to predict the prognosis of MpBC patients [13, 14], which have been proven to have good accuracy. However, for older women with MpBC, the cause of death is not just cancer but many other factors, such as cardiovascular disease. The current nomogram only analyzes the risk factors of cancer patient death, ignoring the competitive risk death factors. The competitive risk model considered other causes of death when developing predictive tools and developed a predictive tool for cancer deaths [15] with greater accuracy and reliability. Wang et al. [15] developed a competitive risk model to predict the cancer-specific survival of older patients with renal cell carcinoma, and it demonstrated good predictive performance, serving as an effective clinical tool. However, currently, there is no competitive risk model available for predicting cancer-specific survival in older women with MpBC. Furthermore, it remains unknown whether the competitive risk model can be applicable to older women with MpBC. Therefore, this study follows the method of Wang et al. to establish a competitive risk model for predicting cancer-specific survival in older women with MpBC. The aim is to accurately predict patients’ survival outcomes and provide a rational basis for individualized treatment.

## Materials and methods

### Data resources

Data on patients diagnosed with MpBC from 2010 to 2019 are from the Surveillance, Epidemiology and End Results (SEER) program. The SEER database is a population-based cancer database in the United States, including 18 cancer registries, covering about 30% of the US population. Since the patient’s personal information in the SEER database cannot be identified, this study does not require ethical approval and informed consent. All the research methods in this study comply with the provisions of the SEER database.

**Fig. 1:**
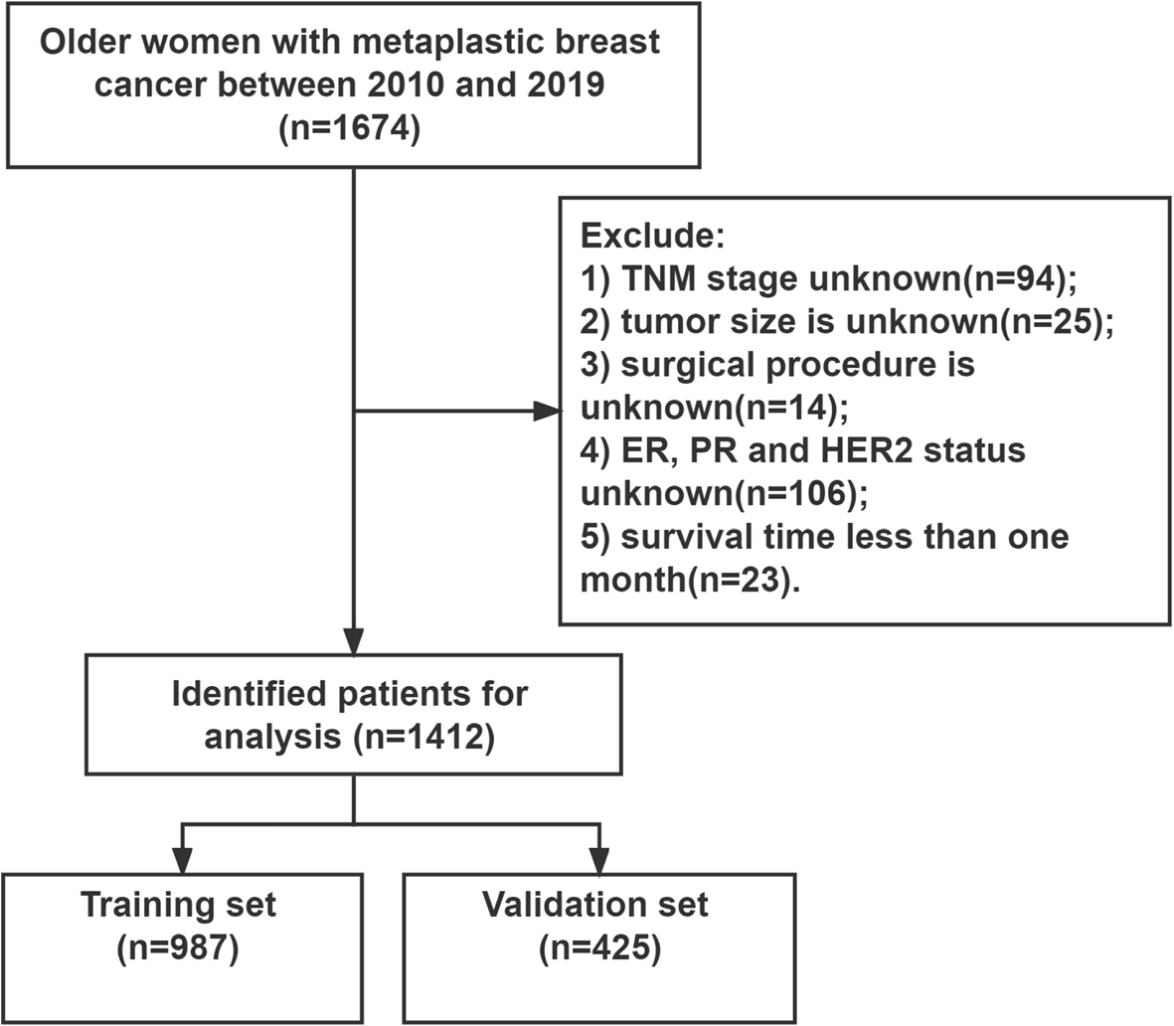
The flow chart of patient selection

This study collected data from older women with MpBC. Inclusion criteria: 1) the diagnosis year is 2010–2019; 2) age ≥ 65 years old; 3) women; 4) the pathological diagnosis was MpBC. Exclusion criteria: 1) TNM stage unknown; 2) tumor size is unknown; 3) surgical procedure is unknown; 4) estrogen receptor(ER), progesterone receptor(PR) and human epidermal growth factor receptor-2(HER2) status unknown; 5) survival time less than one month. The patient screening process is shown in Fig. 1.

### Variables definition

The clinicopathological information of patients, including age, race, marriage, histological type, histological grade, TNM stage, ER, PR and HER2 status, tumor size, and treatment (surgery, radiotherapy and chemotherapy), were obtained from the SEER database. The race is divided into white, black and others (American Indian/AK Native, Asian/Pacific Islander). Marriage is divided into married or unmarried. Histological types included metaplastic carcinoma, squamous cell carcinoma, spindle cell carcinoma, adenosquamous carcinoma and adenocarcinoma mixed. Histological grades, including grades I-IV, were highly differentiated, moderately differentiated, poorly differentiated and undifferentiated, respectively. Surgery is divided into surgery and non-surgery. Radiotherapy and chemotherapy are also divided into yes or No. ER, PR and HER2 status were classified as negative or positive. Patients’ survival status is divided into survival, death from cancer, or death from other causes.

Among these variables, age and tumor size are treated as continuous variable inputs to the model, while marital status, tumor laterality, pathological type, histological grade, TNM stage, surgery, radiation therapy, chemotherapy, ER status, PR status, and HER2 status are treated as categorical variable inputs to the model.

### Development and validation of the competitive risk model

In order to ensure the accuracy of model validation, we divided the data into two relatively independent datasets. All patients were randomly assigned to the training set (70%) and validation set (30%). In the training set, the proportional sub-distribution risk model proposed by Fine and Gray [15] was used to analyze the risk factors affecting patient death. The competitive risk model is closer to reality because it considers other competing endpoints when calculating endpoint events [16]. We also analyzed the risk factors for cancer-specific mortality (CSM) and other causes of mortality (OCM) in older women with MpBC. Based on the risk factors for CSM in patients, we constructed a competitive risk model to predict 1-, 3-, and 5-year cancer-specific survival in patients. Our study methodology was similar to a previous study [17]. When training the model, we utilized a threshold of *P* < 0.05 and performed stepwise regression to identify the best fitting variables, thus constructing a competitive risk model. Breast cancer recurrence in most cases occurs within 1–3 years after diagnosis, and patients who have not experienced a recurrence after 5 years are generally considered clinically cured. Therefore, it is important for us to focus on the 1-year, 3-year, and 5-year survival of older women with MpBC to develop better follow-up strategies. Then we used the concordance index (C-index), the calibration curve and the area under the receiver operating characteristic curve (AUC) to validate the discrimination and accuracy of the model in the training set and the validation set.

### Clinical utility

Decision Curve Analysis (DCA) is a new algorithm to calculate the net profit under different thresholds. DCA was used to calculate the potential clinical value of the new model and compared it with the traditional TNM staging system. In addition, according to the risk value calculated by the competitive risk model, the patients were divided into a high-risk group and a low-risk group by using the Youden index of the receiver operating characteristic curve (ROC) to take the best cut-offcut-off value. Kaplan–Meier (K-M) curve and Log-rank test were used to analyze the survival difference between the low-risk and high-risk groups.

### Statistical analysis

All statistical methods were analyzed by R software 4.1.0 and SPSS 26.0. Continuous variables were described by mean and standard deviation, and a nonparametric U test was used to compare differences between groups. Categorical variables were described by frequency, and chi-square tests were used to compare group differences. The proportional sub-distribution hazard model analyzed the risk factors of CSM and OCM. The Log-rank test and Kaplan–Meier curve analyzed the survival difference between groups. A *p*-value less than 0.05 was considered statistically significant.

## Results

### Patient baseline characteristics

A total of 1412 older women with MpBC were included in this study. Patients were randomly divided into a training set (*n* = 987) and a validation set (*n* = 425). The average age of the patients in this study was 76.0 ± 7.72 years, of which 1145 ( 81.1%) were white, and 591 ( 41.9%) were married. There were 1172 (83.0%) patients with metaplastic carcinoma and 865 ( 61.3%) patients with grade III tumors. There were 695 ( 49.2%) patients of T2 tumors, 1148 ( 81.3%) patients of N0 tumors, and 1348 ( 95.5%) patients of M0 tumors. There were 1339 ( 94.8%) patients receiving surgery, 550 (39.0%) patients receiving radiotherapy, and 613 ( 43.4%) patients receiving chemotherapy. The mean tumor size was 38.5 ± 29.6 mm. There were 1251 (88.6%) PR-negative patients, 1115 (79.0%) ER-negative patients and 1360(96.3%) HER2-negative patients. At present, 903 (64.0%) patients survived, 327 (23.2%) patients died of cancer, and 182 (12.9%) patients died of other causes. The mean survival time of all patients was 38.5 ± 32.0 months. The demographic and clinicopathological characteristics of the patients are shown in Table 1. There was no significant difference between the training set and the validation set.

**Table 1:** Clinicopathological characteristics of older women with metaplastic breast cancer

	**ALL** ***N* = 1412**	**Training set** ***N* = 987**	**Validation set** ***N* = 425**	***P***
Age	76.0 (7.72)	75.9 (7.71)	76.1 (7.75)	0.681
Race				0.988
white	1145 (81.1%)	801 (81.2%)	344 (80.9%)	
black	163 (11.5%)	114 (11.6%)	49 (11.5%)	
other	104 (7.37%)	72 (7.29%)	32 (7.53%)	
Marital				0.849
No	821 (58.1%)	576 (58.4%)	245 (57.6%)	
Married	591 (41.9%)	411 (41.6%)	180 (42.4%)	
Laterality				0.438
Left	727 (51.5%)	501 (50.8%)	226 (53.2%)	
Right	685 (48.5%)	486 (49.2%)	199 (46.8%)	
Histology				0.394
Metaplastic carcinoma	1172 (83.0%)	826 (83.7%)	346 (81.4%)	
Squamous cell carcinoma	75 (5.31%)	51 (5.17%)	24 (5.65%)	
Spindle cell carcinoma	72 (5.10%)	43 (4.36%)	29 (6.82%)	
Adenosquamous carcinoma	38 (2.69%)	27 (2.74%)	11 (2.59%)	
Adenocarcinoma mixed	55 (3.90%)	40 (4.05%)	15 (3.53%)	
Grade				0.943
I	61 (4.32%)	41 (4.15%)	20 (4.72%)	
II	241 (17.1%)	173 (17.5%)	68 (16.0%)	
III	865 (61.3%)	602 (61.0%)	263 (62.0%)	
IV	34 (2.41%)	23 (2.33%)	11 (2.59%)	
Unknown	210 (14.9%)	148 (15.0%)	62 (14.6%)	
T				0.513
T1	377 (26.7%)	261 (26.4%)	116 (27.3%)	
T2	695 (49.2%)	491 (49.7%)	204 (48.0%)	
T3	219 (15.5%)	157 (15.9%)	62 (14.6%)	
T4	121 (8.57%)	78 (7.90%)	43 (10.1%)	
N				0.627
N0	1148 (81.3%)	804 (81.5%)	344 (80.9%)	
N1	192 (13.6%)	137 (13.9%)	55 (12.9%)	
N2	47 (3.33%)	29 (2.94%)	18 (4.24%)	
N3	25 (1.77%)	17 (1.72%)	8 (1.88%)	
M				0.623
M0	1348 (95.5%)	940 (95.2%)	408 (96.0%)	
M1	64 (4.53%)	47 (4.76%)	17 (4.00%)	
Tumor size	38.5 (29.6)	38.0 (27.6)	39.6 (34.0)	0.387
Surgery				0.689
No	73 (5.17%)	49 (4.96%)	24 (5.65%)	
Yes	1339 (94.8%)	938 (95.0%)	401 (94.4%)	
Chemotherapy				0.412
No	799 (56.6%)	551 (55.8%)	248 (58.4%)	
Yes	613 (43.4%)	436 (44.2%)	177 (41.6%)	
Radiation				0.402
No	862 (61.0%)	595 (60.3%)	267 (62.8%)	
Yes	550 (39.0%)	392 (39.7%)	158 (37.2%)	
PR				0.861
Negative	1251 (88.6%)	873 (88.4%)	378 (88.9%)	
Positive	161 (11.4%)	114 (11.6%)	47 (11.1%)	
ER				0.875
Negative	1115 (79.0%)	781 (79.1%)	334 (78.6%)	
Positive	297 (21.0%)	206 (20.9%)	91 (21.4%)	
HER2				0.723
Negative	1360 (96.3%)	949 (96.1%)	411 (96.7%)	
Positive	52 (3.68%)	38 (3.85%)	14 (3.29%)	
Status				0.361
Alive	903 (64.0%)	632 (64.0%)	271 (63.8%)	
Dead Of Cancer	327 (23.2%)	221 (22.4%)	106 (24.9%)	
Dead Of other casuse	182 (12.9%)	134 (13.6%)	48 (11.3%)	
Survival months	38.5 (32.0)	37.9 (31.3)	40.0 (33.5)	0.266

### Prognostic factors of survival

In the training set, the proportional sub-distribution hazard model was used to analyze the risk factors of CSM and OCM. The results showed that age, T stage, N stage, M stage, tumor size, surgery and radiotherapy were risk factors for CSM. In addition, age is a risk factor for OCM in older women with MpBC (Table 2**, **Table S1).

**Table 2:** The proportional sub-distribution risk model predict cancer-specific mortality older women with metaplastic breast cancer

	**Unadjusted model**				**Adjusted model**			
	**HR**	**95%CI**		***P***	**HR**	**95%CI**		***P***
Age	1.03	1.02	1.05	< 0.001	1.024	1.002	1.046	0.03
Race								
white	Reference				Reference			
black	1.289	0.891	1.86	0.18	1.295	0.869	1.929	0.2
other	0.969	0.58	1.62	0.9	1.106	0.646	1.894	0.7
Marital								
No	Reference				Reference			
Married	0.568	0.428	0.753	< 0.001	0.927	0.673	1.276	0.6
Laterality								
Left	Reference				Reference			
Right	0.72	0.552	0.938	0.015	0.877	0.659	1.166	0.4
Histology								
Metaplastic carcinoma	Reference				Reference			
Squamous cell carcinoma	0.883	0.477	1.64	0.69	0.695	0.362	1.333	0.27
Spindle cell carcinoma	1.166	0.594	2.29	0.66	1.879	0.931	3.791	0.078
Adenosquamous carcinoma	0.496	0.191	1.29	0.15	0.458	0.12	1.743	0.25
Adenocarcinoma mixed	0.62	0.252	1.53	0.3	0.581	0.22	1.536	0.27
Grade								
I	Reference				Reference			
II	1.37	0.581	3.21	0.47	1.204	0.514	2.817	0.67
III	1.96	0.886	4.36	0.097	1.415	0.63	3.179	0.4
IV	1.29	3.363	4.58	0.69	0.874	0.235	3.251	0.84
Unknown	2.43	1.05	5.62	0.038	1.524	0.654	3.549	0.33
T								
T1	Reference				Reference			
T2	1.77	1.16	2.7	< 0.001	1.388	0.876	2.198	0.16
T3	5.95	3.86	9.18	< 0.001	2.947	1.564	5.553	0.001
T4	7.84	4.78	12.84	< 0.001	2.89	1.436	5.815	0.003
N								
N0	Reference				Reference			
N1	2.15	1.53	3.01	< 0.001	1.414	0.958	2.086	0.08
N2	3.23	1.91	5.47	< 0.001	1.914	1.105	3.317	0.02
N3	2.76	1.35	5.64	< 0.001	1.468	0.549	3.925	0.4
M								
M0	Reference				Reference			
M1	10.6	6.97	16.2	< 0.001	4.698	2.869	7.692	< 0.001
Tumor size	1.02	1.01	1.02	< 0.001	1.005	0.999	1.012	0.037
Surgery								
No	Reference				Reference			
Yes	0.253	0.158	0.406	< 0.001	0.391	0.233	0.658	< 0.001
Chemotherapy								
No	Reference				Reference			
Yes	0.884	0.678	1.15	0.36	1.039	0.735	1.469	0.8
Radiation								
No	Reference				Reference			
Yes	0.571	0.43	0.758	< 0.001	0.689	0.502	0.947	0.02
PR								
Negative	Reference				Reference			
Positive	0.689	10.436	1.09	0.11	0.82	0.48	1.401	0.5
ER								
Negative	Reference				Reference			
Positive	0.875	0.632	1.21	0.42	0.901	0.607	1.336	0.6
HER2								
Negative	Reference				Reference			
Positive	0.972	0.482	1.96	0.9	0.981	0.415	2.318	0.9

### Development of the competitive risk model

**Fig. 2:**
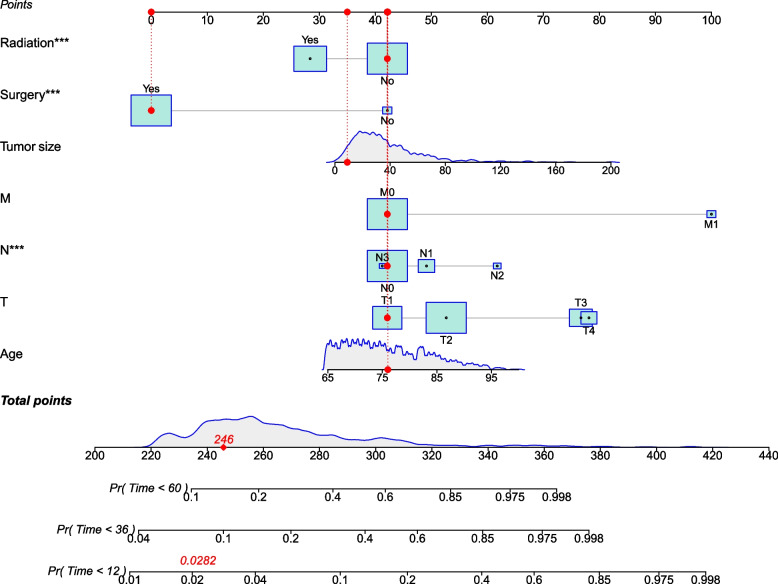
Competitive risk model nomogram for predicting the 1-, 3-, and 5-year cancer-specific survival of MpBC

Based on the proportional sub-distribution hazard model analysis results, we incorporated risk factors affecting CSM in patients. We established a competitive risk model to predict 1-, 3-, and 5-year cancer-specific survival in older women with MpBC. As shown in Fig. 2, older patients have a higher risk of death. The higher the TNM stage and the larger tumors, the greater the risk of death in patients. Patients undergoing surgery and radiotherapy have a higher survival rate.

### Validation of the competitive risk model

**Fig. 3:**
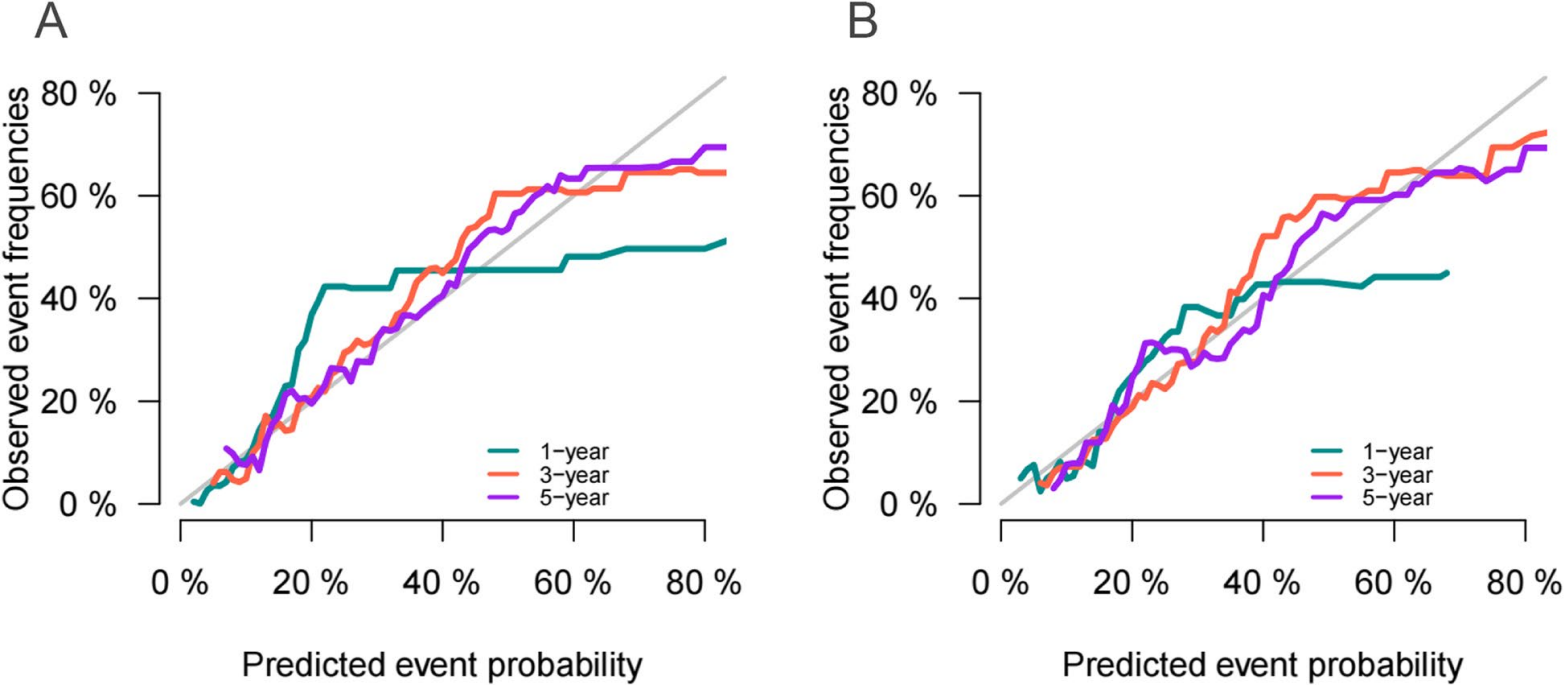
The calibration curves for predicting the cancer-specific survival of older women with MpBC in the training set (**A**) and validation set (**B**)

**Fig. 4:**
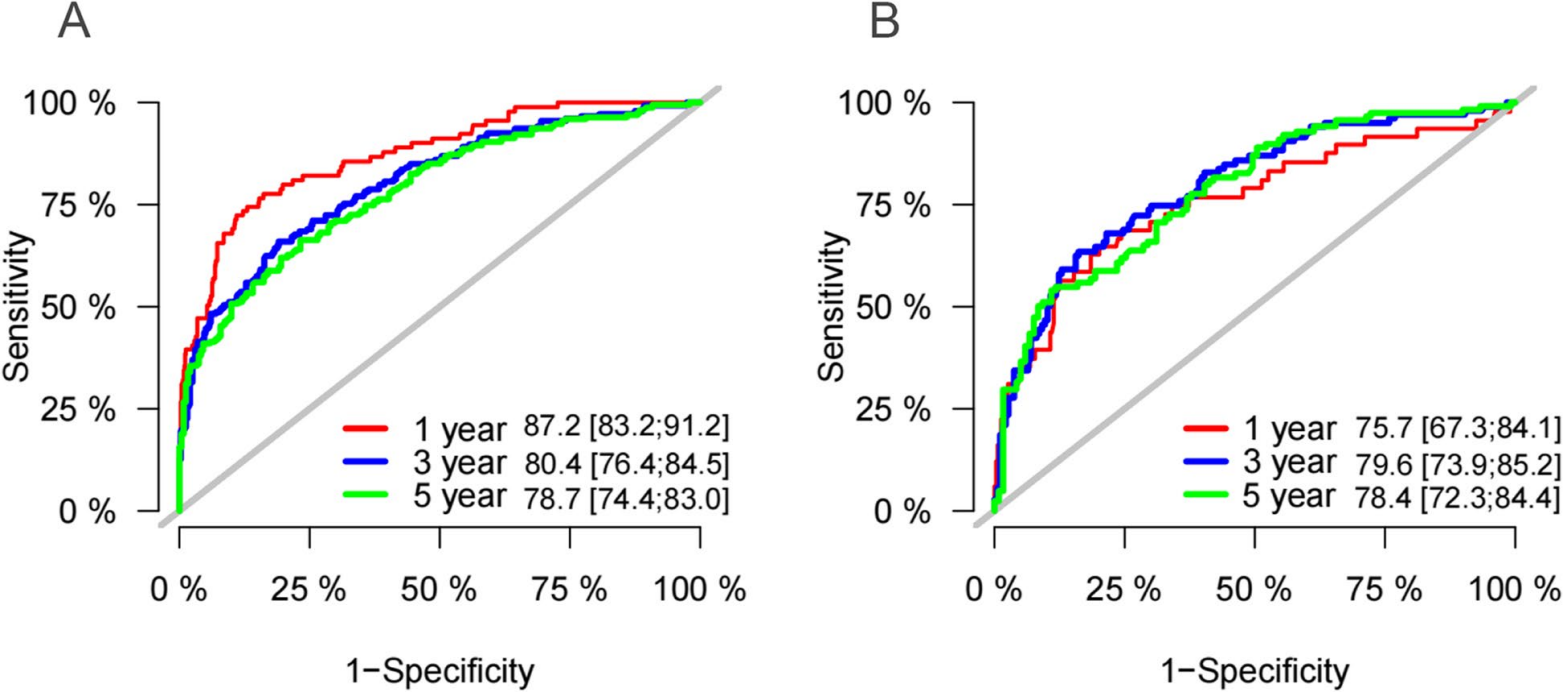
ROC curve with AUC for cancer-specific survival in older women with MpBC. **A** 1-, 3-, and 5-year cancer-specific survival rate in the training set, **B** 1-, 3-, and 5-year cancer-specific survival rate in the validation set

We used a series of validation methods to validate the accuracy and discrimination of the model. The C-index of the model was 0.792 ( 95% CI: 0.763–0.821) in the training set and 0.744 ( 95% CI: 0.691–0.797) in the validation set. The calibration curves in the training and validation sets showed that the model’s predicted values were almost consistent with the actual observed values, which proved that the prediction model had good accuracy (Fig. 3). The AUC results showed that the AUC of the model for predicting 1-, 3-, and 5-year survival in the training set was 87.2, 80.4, and 78.7, respectively. In the validation set, the AUC of the model for predicting 1-, 3-, and 5-year survival were 75.7, 79.6, and 78.4, respectively (Fig. 4). These results show that the prediction model has good accuracy. Furthermore, we compared the C-index and AUC of the nomogram with the traditional TNM staging (Table S2), and the results showed that the nomogram demonstrated superior predictive performance compared to TNM staging.

### Clinical application

**Fig. 5:**
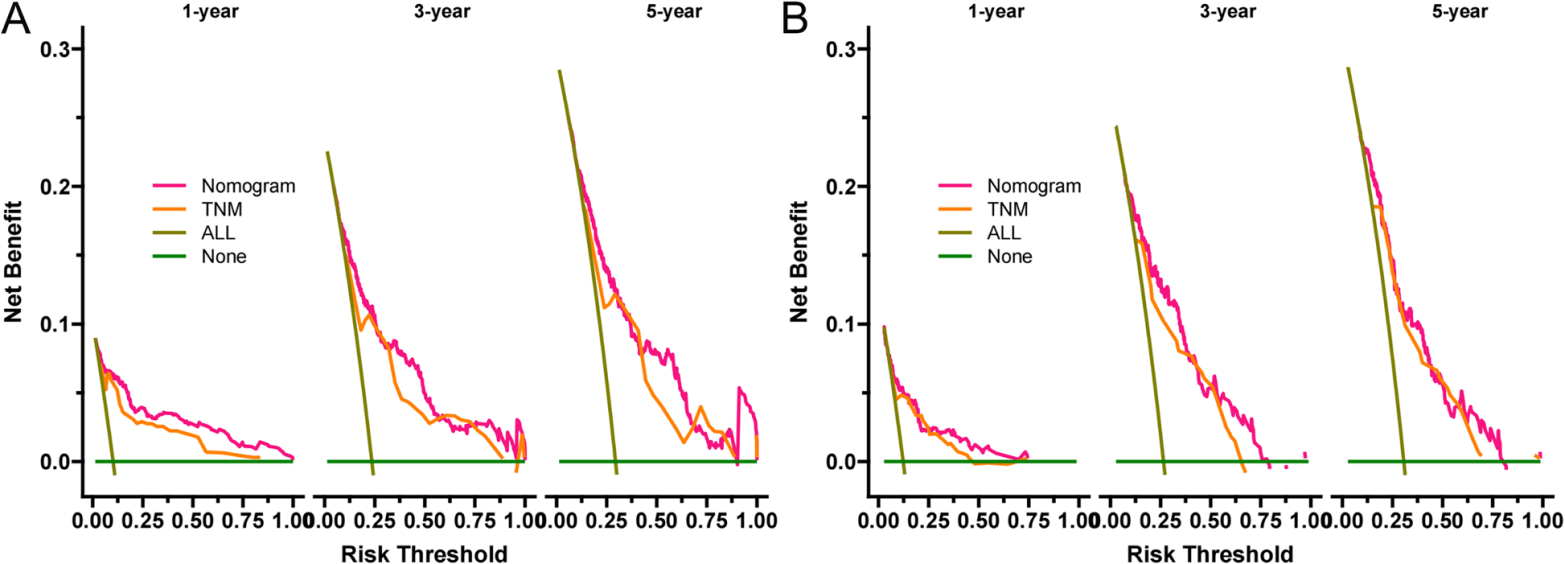
DCA of competitive risk model in the training and validation sets. **A** 1-, 3-, and 5-year cancer-specific survival rate in the training set, **B** 1-, 3-, and 5-year cancer-specific survival rate in the validation set

**Fig. 6:**
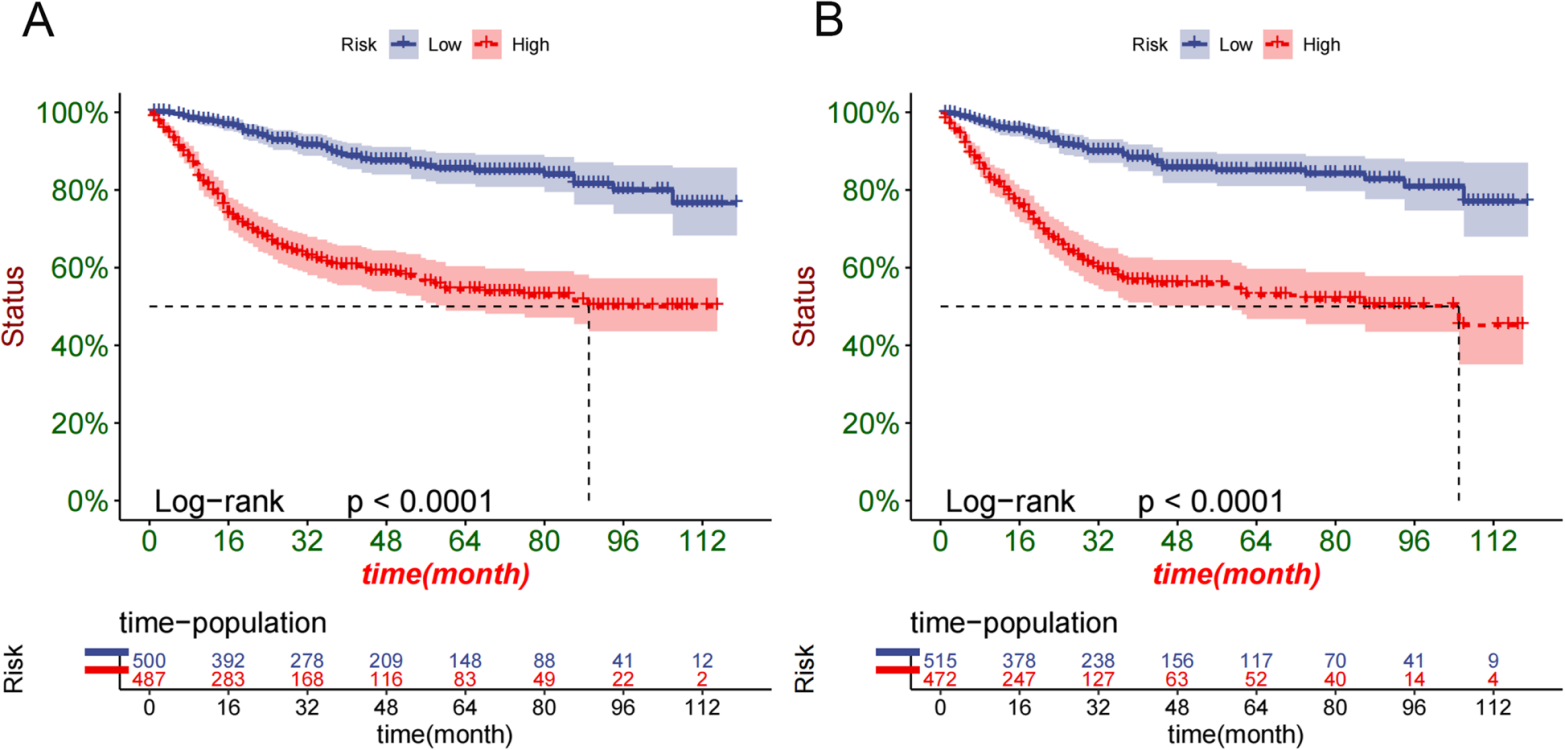
K-M curves of older women with MpBC in training sets (**A**) and validation sets (**B**)

We used DCA to validate the practical clinical value of the model. The results showed that the prediction model showed good practical value in predicting 1-, 3-, and 5-year survival. And the predictive ability of the prediction model is better than the traditional TNM staging system (Fig. 5). Subsequently, we used the established prediction model to calculate the risk value for each patient and used the ROC best cut-off value (85.1) to divide the patients into high-risk groups (≥ 85.1) and low-risk groups (< 85.1). The high-risk group’s 1-, 3-, and 5-year survival rates were 8.15, 6.03 and 5.46%, respectively. The survival rates of low-risk patients were 96.6%, 89.9% and 84.8%, respectively. The K-M curve showed that the survival rate of patients in the high-risk group was significantly lower than that in the low-risk group (Fig. 6).

## Discussion

MpBC is mainly composed of mesenchymal histological components and epithelial cells, a rare breast cancer type [18]. MpBC is a highly malignant breast cancer divided into low-grade and high-grade MpBC according to histopathological features. Low-grade MpBC includes fibroma-like MpBC and adenosquamous carcinoma [19]. High-grade MpBC includes spindle cell carcinomas, squamous cell carcinoma, and pleomorphic mesenchymal differentiation MpBC. Previous studies have found that more than 90% of MpBC hormone receptors and HER2 are negative [20]. Since MpBC is a rare type of breast cancer, studies on its clinical features and prognostic factors are minimal. Currently, no clinical diagnosis and treatment guidelines or prognostic factors to explore are widely recognized worldwide. The prognosis of MpBC is worse than that of common triple-negative breast cancer. Therefore, it is necessary further to explore the prognostic factors of MpBC [21].

Older breast cancer patients are a particular group. Compared with young women, they have different physiology, tumor biological behaviour and social dynamics [22–24]. Most older women with breast cancer are low-grade and hormone receptor-positive, so they often receive non-surgical treatment. However, older women with MpBC will face the challenge of hormone receptor and HER2 negative, so their treatment will be different. In addition, the treatment of older women with MpBC must be personalized, as they also face a competitive risk of death from diseases other than cancer. Older patients’ comorbidities and decreased physiological status may prevent them from benefiting from treatment as young patients do [11, 25]. Different social support systems will also affect the treatment experience of older patients. The lack of independence of the older will lead to the obstruction of compliance with drugs and appointments.

In this study, we reported the clinicopathological features and prognostic factors of older women with MpBC in the SEER database. This study found that marital status and race were not factors affecting cancer-specific survival in older women with MpBC. The pathological subtype of MpBC was also not significantly correlated with cancer-specific survival. Previous studies have shown that the lymph node metastasis rate of MpBC patients is about 22–31% [26]. Our study found that the lymph node metastasis rate of older women with MpBC is less than 20%, slightly lower than that of overall MpBC patients. Moreover, our study found that lymph node metastasis is a risk factor for cancer-specific survival in older women with MpBC, similar to the study of Lee et al. [3].

A previous study has reported that chemotherapy can improve the prognosis of MpBC patients [27]. However, another study has shown that MpBC has a minimal effect on chemotherapy due to chemotherapy resistance [28]. Rakha et al. also found that chemotherapy can only prolong the survival of patients with early disease [29]. Our study also confirmed that chemotherapy had no significant benefit for cancer-specific survival in older women with MpBC. Because MpBC patients lack standard treatment options, radiotherapy is also controversial. A study has shown that radiotherapy can reduce the risk of local recurrence of breast cancer and residual lesions after surgery [30]. Our study shows that radiotherapy can significantly benefit older women with MpBC. In addition, we found that most patients underwent surgery and that patients who underwent surgery significantly improved survival rates, similar to a previous study [31]. Therefore, surgery and radiotherapy are critical for older patients with MpBC.

This study found that age, T stage, N stage, M stage, tumor size, surgery and radiotherapy were risk factors for cancer-specific survival in older patients with MpBC. We developed a competitive risk model nomogram based on these variables to predict 1-, 3-, and 5-year cancer-specific survival in older women with MpBC. The competitive risk model is a nomogram and a simple prediction tool. By accurately predicting the survival time of patients, it can help doctors make clinical decisions and improve patient compliance.

There are still some limitations in this study. First, the study lacked essential variables such as comorbidity, chemotherapy regimen, endocrine therapy, radiation dose, and physiological status. However, this study included essential variables, such as tumor staging and treatment. Therefore, the prediction tool has good clinical value. Secondly, this study is retrospective, and there is still a selection bias that is difficult to adjust. Therefore, further prospective studies to confirm the study results are necessary. Finally, this study’s competitive risk prediction model only conducted internal cross-validation. Although the model was confirmed to have good accuracy, further external verification is still needed.

## Conclusion

This study explored the competitive risk factors for cancer-specific death in older women with MpBC. Age, T stage, N stage, M stage, tumor size, surgery and radiotherapy were risk factors for cancer-specific death in older patients with MpBC. Based on these risk factors, we developed a competitive risk model to predict cancer-specific survival in older women with MpBC. The validation results of the model show that it is a very effective and reliable prediction tool. This predictive tool allows doctors and patients to make individualized clinical decisions.

## Supplementary Information

**Additional file 1: Table S1.** The proportional sub-distribution risk model predict other causes mortality older women with metaplastic breast cancer.

**Additional file 2: Table S2.** C-index and AUC in the training and validation sets.

## Data Availability

The data analyzed in this study is available at https://seer.Cancer.gov/.
