# Supplementary material for: Development and validation of competitive risk model for older women with metaplastic breast cancer
Source: BMC Womens Health. 2023 Jul 14;23:374. doi: 10.1186/s12905-023-02513-x (PMC10349515; doi:10.1186/s12905-023-02513-x)
Supplement: Supplementary file 1 — Additional file 1: Table S1. The proportional sub-distribution risk model predict other causes mortality older women with metaplastic breast cancer. [file 12905_2023_2513_MOESM1_ESM.docx]

**Table S1. The proportional sub-distribution risk model predict other causes mortality older women with metaplastic breast cancer.**

|  | **Unadjusted model** | | | |  | **Adjusted model** | | | |
| --- | --- | --- | --- | --- | --- | --- | --- | --- | --- |
|  | **HR** | **95%CI** | | **P** |  | **HR** | **95%CI** | | **P** |
| Age | 1.1 | 1.07 | 1.12 | <0.001 |  | 1.086 | 1.0582 | 1.12 | <0.001 |
| Race |  |  |  |  |  |  |  |  |  |
| white | Reference |  |  |  |  | Reference |  |  |  |
| black | 0.893 | 0.514 | 1.55 | 0.69 |  | 1.126 | 0.623 | 2.03 | 0.7 |
| other | 0.842 | 0.418 | 1.7 | 0.63 |  | 0.805 | 0.3775 | 1.72 | 0.6 |
| Marital |  |  |  |  |  |  |  |  |  |
| No | Reference |  |  |  |  | Reference |  |  |  |
| Married | 0.72 | 0.506 | 1.02 | 0.068 |  | 1.11 | 0.7616 | 1.62 | 0.6 |
| Laterality |  |  |  |  |  |  |  |  |  |
| Left | Reference |  |  |  |  | Reference |  |  |  |
| Right | 1.01 | 0.723 | 1.42 | 0.95 |  | 0.974 | 0.6852 | 1.38 | 0.9 |
| Histology |  |  |  |  |  |  |  |  |  |
| Metaplastic carcinoma | Reference |  |  |  |  | Reference |  |  |  |
| Squamous cell carcinoma | 2.3 | 1.347 | 3.92 | 0.002 |  | 2.17 | 1.2595 | 3.74 | 0.005 |
| Spindle cell carcinoma | 1.45 | 0.68 | 3.07 | 0.34 |  | 1.156 | 0.5339 | 2.5 | 0.7 |
| Adenosquamous carcinoma | 1.98 | 0.918 | 4.28 | 0.08 |  | 2.911 | 1.1303 | 7.49 | 0.03 |
| Adenocarcinoma mixed | 1.29 | 0.522 | 3.21 | 0.58 |  | 0.876 | 0.3174 | 2.42 | 0.8 |
| Grade |  |  |  |  |  |  |  |  |  |
| I | Reference |  |  |  |  | Reference |  |  |  |
| II | 0.549 | 0.227 | 1.33 | 0.19 |  | 0.792 | 0.3193 | 1.97 | 0.6 |
| III | 0.967 | 0.453 | 2.06 | 0.93 |  | 1.685 | 0.6867 | 4.14 | 0.3 |
| IV | 1.046 | 0.321 | 3.41 | 0.94 |  | 3.196 | 0.8682 | 11.77 | 0.08 |
| Unknown | 0.949 | 0.406 | 2.22 | 0.9 |  | 1.495 | 0.5403 | 4.13 | 0.4 |
| T |  |  |  |  |  |  |  |  |  |
| T1 | Reference |  |  |  |  | Reference |  |  |  |
| T2 | 0.929 | 0.621 | 1.39 | 0.72 |  | 0.763 | 0.446 | 1.3 | 0.3 |
| T3 | 1.191 | 0.719 | 1.97 | 0.5 |  | 0.904 | 0.3241 | 2.52 | 0.9 |
| T4 | 0.734 | 0.34 | 1.58 | 0.43 |  | 0.908 | 0.2953 | 2.79 | 0.9 |
| N |  |  |  |  |  |  |  |  |  |
| N0 | Reference |  |  |  |  | Reference |  |  |  |
| N1 | 0.504 | 0.269 | 0.945 | 0.033 |  | 0.618 | 0.2869 | 1.33 | 0.2 |
| N2 | 0.607 | 0.187 | 1.975 | 0.4 |  | 0.697 | 0.2225 | 2.18 | 0.5 |
| N3 | 1.071 | 0.346 | 3.314 | 0.9 |  | 1.49 | 0.4152 | 5.35 | 0.5 |
| M |  |  |  |  |  |  |  |  |  |
| M0 | Reference |  |  |  |  | Reference |  |  |  |
| M1 | 0.281 | 0.068 | 1.17 | 0.08 |  | 0.301 | 0.0679 | 1.34 | 0.1 |
| Tumor size | 0.999 | 0.993 | 0.01 | 0.78 |  | 0.997 | 0.982 | 1.01 | 0.7 |
| Surgery |  |  |  |  |  |  |  |  |  |
| No | Reference |  |  |  |  | Reference |  |  |  |
| Yes | 0.714 | 0.337 | 1.51 | 0.38 |  | 0.843 | 0.337 | 2.11 | 0.7 |
| Chemotherapy |  |  |  |  |  |  |  |  |  |
| No | Reference |  |  |  |  | Reference |  |  |  |
| Yes | 0.314 | 0.208 | 0.474 | P<0.001 |  | 0.651 | 0.3952 | 1.07 | 0.09 |
| Radiation |  |  |  |  |  |  |  |  |  |
| No | Reference |  |  |  |  | Reference |  |  |  |
| Yes | 0.587 | 0.407 | 0.846 | 0.004 |  | 0.838 | 0.5586 | 1.26 | 0.4 |
| PR |  |  |  |  |  |  |  |  |  |
| Negative | Reference |  |  |  |  | Reference |  |  |  |
| Positive | 0.752 | 0.427 | 1.32 | 0.32 |  | 0.752 | 0.4101 | 1.38 | 0.4 |
| ER |  |  |  |  |  |  |  |  |  |
| Negative | Reference |  |  |  |  | Reference |  |  |  |
| Positive | 1.03 | 0.684 | 1.55 | 0.89 |  | 0.974 | 0.6119 | 1.55 | 0.9 |
| HER2 |  |  |  |  |  |  |  |  |  |
| Negative | Reference |  |  |  |  | Reference |  |  |  |
| Positive | 0.645 | 0.254 | 1.64 | 0.36 |  | 0.472 | 0.1646 | 1.35 | 0.2 |
