# Supplementary material for: Development and validation of competitive risk model for older women with metaplastic breast cancer
Source: BMC Womens Health. 2023 Jul 14;23:374. doi: 10.1186/s12905-023-02513-x (PMC10349515; doi:10.1186/s12905-023-02513-x)
Supplement: Supplementary file 2 — Additional file 2: Table S2. C-index and AUC in the training and validation sets. [file 12905_2023_2513_MOESM2_ESM.docx]

**Table S2. C-index and** **AUC in the training and** **validation sets.**

|  | **Training set** | |  | **Validation set** | |
| --- | --- | --- | --- | --- | --- |
| **C-index** |  |  |  |  |  |
| Nomogram | 0.792 | 0.763-0.821 |  | 0.744 | 0.691-0.797 |
| TNM | 0.75 | 0.717-0.783 |  | 0.716 | 0.663-0.769 |
| **AUC** |  |  |  |  |  |
| Nomogram 1-year | 87.2 | 83.2-91.2 |  | 75.7 | 67.3-84.1 |
| Nomogram 3-year | 80.4 | 76.4-84.5 |  | 79.6 | 73.9-85.2 |
| Nomogram 5-year | 78.7 | 74.4-83.0 |  | 78.4 | 72.3-84.4 |
| TNM 1-year | 83.1 | 78.4-87.8 |  | 73.4 | 64.9-81.8 |
| TNM 3-year | 76 | 71.7-80.4 |  | 75.9 | 70.1-81.7 |
| TNM 5-year | 73.7 | 69.1-78.3 |  | 76.2 | 70.3-82.2 |
